# Calabar Bean in Spotted Fever

**Published:** 1872-04

**Authors:** 


### Calabar Bean in Spotted Fever.

In our last issue we made reprint of an article in which calabar bean was spoken highly of as a remedy in cerebro-spinal meningitis. Without intending to recommend this drug or give the idea, that it is generally accepted as useful in the treatment of this fearful malady, we still desire to say, that fresh supply has been received by Mr. Wm. Peabody, and that any who desire can take the opportunity to test for themselves its virtues. The following letter we are allowed to copy which fully explains itself.

BROOKLYN, April 10th, 1872.

Mr. W. H. PEABODY, Buffalo.—*Dear Sir.*–The extract calabar bean goes by this mail as ordered. In reply to your inquiry as to what I know of it, dose etc., I must reply that I only know what I read, and that seems to lead to the conclusion that very little that is at all definite has been yet attained, except in eye surgery. It is evidently a potent agency and requires much observation. In tetanus it has not fulfilled the expectations of those who at first spoke highly of it.

This alcoholic extract as sent you is very good and represents the Bean in the proportion of about 2 p. c.. That is 100 lbs Beans yield about 2 lbs extract. I have not known it to be given in more than ¼ grain doses, and it is more frequently given in ⅛ grain doses, and the smaller is the better to begin with. It should however be increased till the effects are obtained. I can hardly credit the large doses lately reported, two or three grains, if the extract was good.

Very respectfully yours, E. R. SQUIBB.

